# Therapeutic misunderstandings in modern research

**DOI:** 10.1111/bioe.13241

**Published:** 2023-12-19

**Authors:** Sarah Heynemann, Wendy Lipworth, Sue‐Anne McLachlan, Jennifer Philip, Tom John, Ian Kerridge

**Affiliations:** ^1^ Sydney Health Ethics, Faculty of Medicine and Health The University of Sydney Sydney New South Wales Australia; ^2^ Department of Philosophy Macquarie University Sydney New South Wales Australia; ^3^ Department of Medical Oncology St Vincent's Hospital Melbourne Victoria Australia; ^4^ Department of Medicine, Faculty of Medicine, Dentistry and Health Sciences The University of Melbourne Melbourne Victoria Australia; ^5^ Department of Palliative Care St Vincent's Hospital Melbourne Victoria Australia; ^6^ Department of Palliative Care Peter MacCallum Cancer Centre Melbourne Victoria Australia; ^7^ Department of Medical Oncology Peter MacCallum Cancer Centre Melbourne Victoria Australia; ^8^ Sir Peter MacCallum Department of Medical Oncology The University of Melbourne Melbourne Victoria Australia; ^9^ Haematology Department Royal North Shore Hospital Sydney New South Wales Australia

**Keywords:** adaptive clinical trial, Bayes theorem, clinical trials, informed consent, research ethics, therapeutic misconception

## Abstract

Clinical trials play a crucial role in generating evidence about healthcare interventions and improving outcomes for current and future patients. For individual trial participants, however, there are inevitably trade‐offs involved in clinical trial participation, given that trials have traditionally been designed to benefit future patient populations rather than to offer personalised care. Failure to understand the distinction between research and clinical care and the likelihood of benefit from participation in clinical trials has been termed the ‘therapeutic misconception’. The evolution of the clinical trials landscape, including greater integration of clinical trials into healthcare and development of novel trial methodologies, may reinforce the significance of the therapeutic misconception and other forms of misunderstanding while at the same time (paradoxically) challenging its salience. Using cancer clinical trials as an exemplar, we describe how methodological changes in early‐ and late‐phase clinical trial designs, as well as changes in the design and delivery of healthcare, impact upon the therapeutic misconception. We suggest that this provides an impetus to re‐examine the ethics of clinical research, particularly in relation to trial access, participant selection, communication and consent, and role delineation.

## 1 INTRODUCTION

It has now been 40 years since the phenomenon termed the ‘therapeutic misconception’ was first described, in the context of evaluation of participants' understanding of a randomised controlled trial (RCT) of a psychotropic agent.^1^ While participants were able to describe, in theory, the nature of research mechanisms such as blinding, randomisation and placebo, there was a clear problem when they were asked to consider the way they would be treated and the likelihood that participation would be orientated towards their own well‐being. When asked how they would receive treatment, one participant responded: ‘I hope it isn't by chance’, going on to express that ‘each participant would probably receive the medication needed’ or personalised care tailored to their own needs, rather than management per trial protocol.^2^ This individual's response captures the central problem to which the therapeutic misconception draws attention—namely, the conflation of clinical research with clinical care, two rather different enterprises with vastly different goals. In this paper, we will consider persistent conceptual challenges surrounding the therapeutic misconception and related misunderstandings. We then go on to highlight some emerging and, as yet, unexamined implications for the therapeutic misconception with the evolution of clinical trial designs and the widespread movement towards seamless integration of clinical research into clinical care. In so doing, this paper seeks to deconstruct a well‐recognised concept within research ethics, contextualise it in the modern clinical trials landscape and offer some preliminary practical suggestions for operationalizing these ideas. We focus particularly on clinical trials for adults with solid cancers.

## 2 THE EVOLUTION OF IDEAS ABOUT THE THERAPEUTIC MISCONCEPTION

When the therapeutic misconception was first described, it alerted both clinicians and researchers to the challenge of the ‘therapeutic orientation’ towards clinical trials^3^ and the difficulties that patients have in understanding the epistemic distinction between standard clinical practice and management received within clinical trials.^4^ While both practices share concern regarding patients' well‐being; routine care seeks to deliver ‘personalised care’,^5^ prioritising the best interests of individuals; whereas research mostly seeks to bring about new, ‘generalisable’,^6^ knowledge for the benefit of future patient populations. There are, therefore, inevitable trade‐offs in research, which need to be considered and accepted by individuals considering trial participation.

There is evidence that, while patients may have many reasons for participating in trials, including altruism, and a sense of obligation to family, their clinicians or institution;^7^ empirical evidence suggests that most are motivated by the possibility of direct medical benefit.^8^ Perceptions among some patients that novel therapies represent superior, ‘cutting edge’ treatment advances have been well‐documented.^9^ However, the reality is that participation in a clinical trial of a novel intervention or therapeutic provides no guarantee of benefit to participants. Furthermore, it is also possible that participants may be disadvantaged or harmed by trial participation, and experience other indirect burdens such as those associated with additional, noncare‐related, testing (e.g., blood draws or tissue biopsies) and increased frequency of hospital attendance. For some patients, such requirements may be perceived as advantageous (e.g., perception of closer monitoring,^10^ albeit debated^11^), while for others there may be Quality of Life (QoL) costs associated with trial participation.^12^ For example, additional trial‐mandated scans may contribute to scan anxiety,^13^ particularly in the case of pseudo‐progression, where an earlier scan suggests cancer progression; however, a subsequent scan notes this not to be the case.^14^ Gupta et al. advocate for reporting of ‘time toxicity’ as a QoL metric in cancer clinical trials, highlighting examples of trials in which small benefits in overall survival may, arguably, be counterbalanced by days spent at the hospital.^15^ These potential costs and burdens are not insignificant, particularly for patients with a serious or incurable disease such as cancer.

Importantly, it is not only patients who sometimes conflate research with clinical care. As Miller and Brody have observed, clinicians and researchers may make the same error,^16^ which may affect both which trials they recommend to patients as well as the ways in which they describe the rationale and processes of research to potential participants. To describe this phenomenon, Green introduced the concept of the ‘therapeutic misdirection’, referencing instances in which physician‐investigators fail to recognise implications of the research‐care demarcation in such a manner as to ‘adversely impact participant welfare or the scientific integrity of the study’.^17^ In this regard, it is worth noting that in a retrospective audit conducted by the same author of Institutional Review Board (IRB) protocol exemption requests at a US centre, among requests to enrol participants on phase I trials who did not meet eligibility criteria, by far the most common justification provided was to ‘allow the patient to benefit by receiving treatment’, which Green suggests underlines a therapeutic orientation adopted by physician‐investigators.^18^

**Figure 1:**
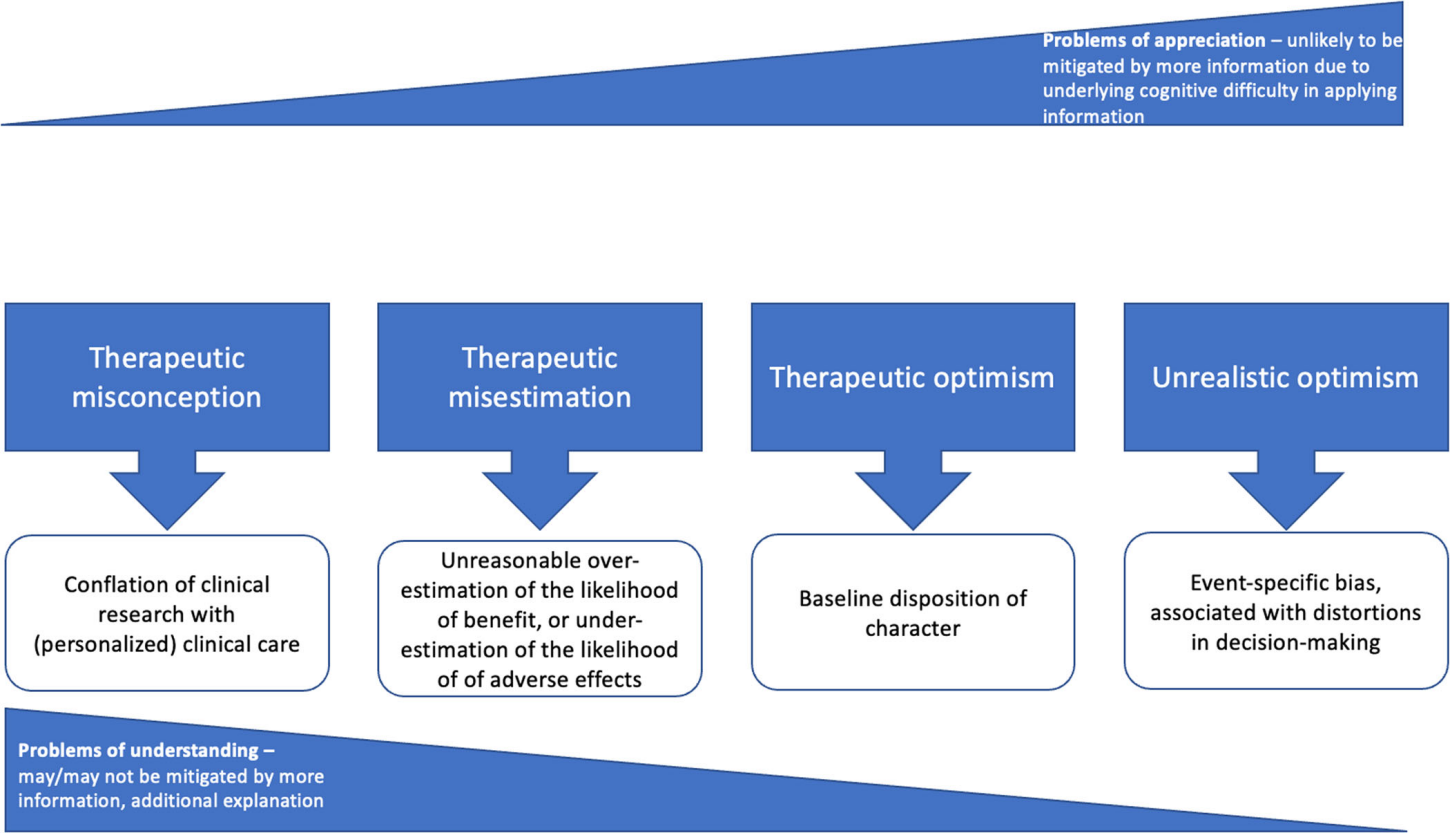
The therapeutic misconception exists along a continuum of potential misunderstandings held by research participants regarding clinical trials. *Source*: Adapted, with authors' permission, from model proposed by Sisk and Kodish (note 22, pp. 13–20).

While the idea of the therapeutic misconception has traditionally focused on misunderstandings of the notion of research as being similar if not equivalent to standard clinical care,^19^ other epistemic errors may also arise. A related concept, the ‘therapeutic misestimation’, has been proposed by Horng and Grady.^20^ This describes circumstances in which research participants either significantly overestimate the likelihood that they will receive benefit from clinical trial participation or, conversely, underestimate the likelihood of risk due to adverse events. According to Horng and Grady, the therapeutic misconception is always ethically problematic^21^ because it represents a categorical error, whereas the ethical import of the therapeutic misestimation is dependent upon the magnitude of the misunderstanding and on the patient's specific circumstances. For example, the therapeutic misestimation may be particularly morally significant for a professional violinist who grossly mis‐underestimates the likelihood of nerve damage to their fingertips (that is, peripheral neuropathy) due to participation in a clinical trial of a novel therapeutic which lists this as a possible adverse event (all the more so if they had the option of proceeding with a routine alternative known *not* to cause this side‐effect). The view that the therapeutic misconception is always morally significant, while the misestimation may not be, is, however, controversial. In an attempt to resolve this issue, Sish and Kodish have advanced the concept of a ‘continuum of misperceptions’,^22^ according to which there are varying degrees of optimism, some of which may also impact upon participant understanding (Figure 1). They build on the empirical work of Jansen and colleagues in noting a distinction between ‘reasonable’ optimism, which may exist as a baseline character disposition, and ‘unrealistic optimism’, an event‐specific cognitive bias which may unhelpfully distort thinking and decision‐making processes.^23^

The therapeutic misestimation may or may not coincide with the therapeutic misconception.^24^ Even if an individual recognises that they are participating in research and not undergoing routine care, misestimations can still occur for other reasons, for example, if the person is subject to ‘unrealistic optimism’.^25^ Some people distinguish between the therapeutic misconception and therapeutic misestimation,^26^ while others view the therapeutic misestimation as a type of therapeutic misconception.^27^ In this article, we use the phrase ‘therapeutic misconception’ to refer to both kinds of misunderstanding, unless we specify otherwise.

Since the initial description of the therapeutic misconception, its theoretical claims have been supported by a large body of empirical evidence, with numerous qualitative and quantitative studies examining evidence for the phenomenon across a variety of participant cohorts, and exploring possible causative factors.^28^ Many of these studies have focused on the Phase I clinical trial setting in adult oncology^29^ (including First‐In‐Human [FIH] trials of novel anti‐cancer therapeutics). Over time, studies of the therapeutic misconception have been refined to ensure that questions seeking to identify evidence of the phenomenon are framed in such a way as to avoid over‐ or underestimation of its occurrence.^30^ Numerous factors that may increase the likelihood of a therapeutic misconception have been described in empirical studies, including lower educational status^31^ and ‘unrealistic’ optimism.^32^ These findings support Appelbaum's notion that research participants may experience difficulties with both perception of ‘the intended meaning of information [provided during the consent process]’ and/or recognition of ‘the implications of this information [for oneself]’.^33^ Thus, while receipt of sufficient, well‐communicated information during the consent process may limit inadequacies of understanding, problems of appreciation may endure.^34^

Over the past four decades, further research has complicated the idea of the therapeutic misconception—for example, questioning what the ‘ideal’ level of understanding should be for consent to be valid, and noting that this may vary between stakeholders (e.g., patient/participant and researcher). It has also been noted that the nature of understanding needed will necessarily vary according to the type and phase of research (e.g., Phase I vs. Phase III RCT) and the nature of the trial methodologic characteristics (e.g., blinding, randomisation). A 2007, multidisciplinary, working party which attempted to establish a consensus definition for the therapeutic misconception concluded that application of the phenomenon would inevitably vary between trials.^35^

## 3 INTEGRATION OF RESEARCH INTO PRACTICE AND EVOLUTION OF CLINICAL TRIAL DESIGNS

The challenge of mapping the boundaries between care and clinical research has long been recognised, including in the Belmont Report published in 1978.^36^ In many respects, such complexity is unsurprising, given that unresolved clinical questions typically form the basis of research questions for clinical trials. In recent years, the clinical trials landscape has changed in such a way that is becoming increasingly difficult to distinguish research from clinical care. In the discussion that follows, we use the case of adult oncology to show how the clinical trials landscape has changed since the initial proposal of the therapeutic misconception—both with respect to (1) the interface between clinical practice and research and (2) clinical trial methodology, across both the early‐ and late‐phase settings. In so doing, we note how such changes both reinforce the significance of the therapeutic misconception and challenge its salience.

The relationship between routine clinical care and clinical trials has become increasingly seamless in oncology, and several factors have contributed to this. First, at a policy level there has been a trend towards (rightly or wrongly) framing of ‘best practice’ clinical care as contingent on integration with trial participation. This is sometimes explicit–for example, the American‐based National Comprehensive Cancer Network® (NCCN®) guidelines state that ‘NCCN® believes that the best management of any patient with cancer is in a clinical trial’.^37^ At other times, calls for integration are more implicit, for example, in the 2015 American Society of Clinical Oncology (ASCO) statement ‘The Critical Role of Phase I Trials in Cancer Research and Treatment’,^38^ or in the slogan ‘Clinical Trial Care is Good Care’^39^ present on the website of Australian comprehensive cancer centre, Peter MacCallum Cancer Centre. Statements such as these may be contextualised amidst a broader cultural shift, beyond oncology, towards the adoption of ‘learning healthcare systems’^40^ approaches, as advocated by the Institute of Medicine in the United States,^41^ in which data and evidence are continually generated, and research outcomes are synthesised and integrated in ‘real‐time’ within healthcare systems.^42^ As Faden and colleagues note, according to a learning health systems approach, ‘it is acceptable and indeed essential to integrate research and practice’, though they further suggest that such positions simultaneously prompt the need for a rethinking of ethical frameworks given departure from both traditional research ethics and clinical ethics norms.^43^ These ideas are implicitly underpinned by the view that patients have an increasing chance of benefiting from trial participation.

This increasingly therapeutic orientation towards clinical trials within cancer care is not, however, without its critics. Such debate, which often centres on Phase I cancer trials, is evident in the diversity of perspectives regarding the scope and goal of such trials, including, for example, ‘a tool for eliminating unpromising treatments before they are committed to later phase trials’,^44^ ‘a tool for signal‐finding and identifying an appropriate patient population for further development’^45^ and ‘a last resort for patients with advanced disease … in some malignancies and some scenarios, … the most promising treatment option’.^46^ Critics of the 2015 ASCO policy statement,^47^ such as Jonathan Kimmelman,^48^ highlight the importance of acknowledging the difference between ‘therapeutic intent’ and ‘therapeutic benefit’, with the latter a question of empirical evidence and, importantly, not necessarily assured by adoption of the former. In reply, former ASCO president Howard Burris seemingly acknowledges this nuance, however maintains the ‘goal of these positions [the ASCO phase I policy statement] is to ensure that patients for whom cancer‐directed therapy is medically appropriate are offered the opportunity to participate in clinical trials of all phases…’.^49^

### 3.1 Recent changes to Phase I trials

**Figure 2:**
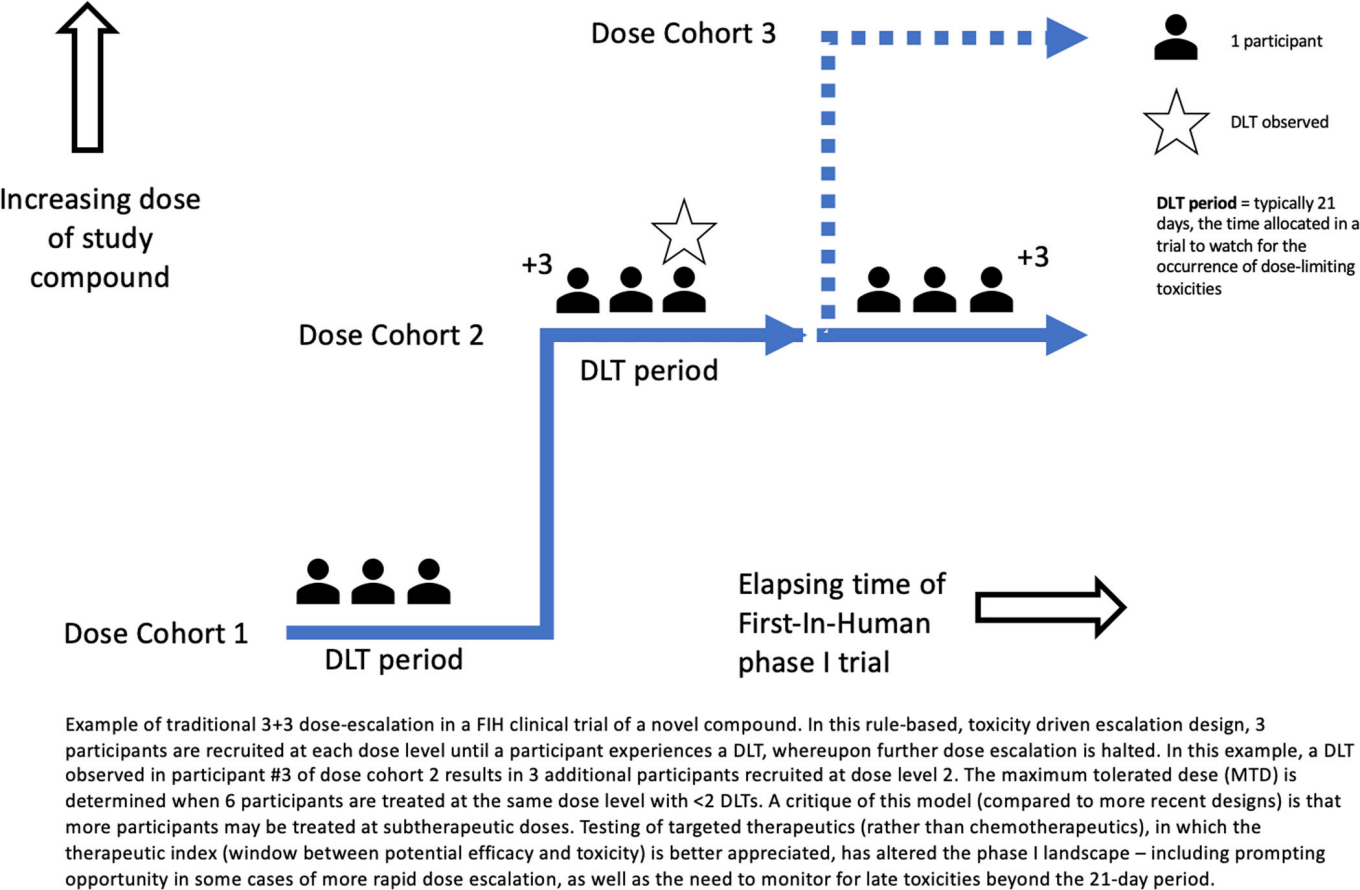
Schematic of standard 3 + 3 dose escalation in a First‐In‐Human Phase I trial of a novel anticancer compound.

Debates about how to frame trial participation have occurred on a background of significant changes in clinical trial design. In Phase I, FIH, cancer clinical trials, traditional practice has been to gradually escalate the dose of a novel compound in small numbers of patients, to determine safety and dosing of novel agents, with determination of efficacy, at most, a secondary objective. Phase I cancer clinical trial participants have, historically, usually been patients with advanced, treatment‐refractory cancers with no further standard‐of‐care treatment options. In a traditional FIH, Phase I trial utilising ‘3 + 3’ dose escalation, three patients are initially recruited at the lowest dose. They are then observed for a minimum period for prespecified dose‐limiting toxicities (DLTs) (Figure 2), after which another three patients are treated at a higher dose until there is suspicion of dose‐related toxicity. While this ‘3 + 3’ approach is cautious^50^ and, historically, has been predicated on the typically narrow therapeutic index of cytotoxic agents, it may also result in a larger proportion of participants receiving what subsequently emerges to be sub‐therapeutic dosing if the eventual maximum tolerated dose (MTD), and recommended phase II dose (RP2D), are much higher.^51^ There have been a number of other recent adaptations to Phase I trial design. These include mechanisms to facilitate greater efficiency and precision in determination of the RP2D (e.g., pharmacokinetically guided dose escalation, guided by blood sampling to assess drug absorption, distribution, metabolism and excretion during the study rather than empiric predictions *a priori*; model‐based designs, such as the continual reassessment method (CRM), in which estimation of the dose‐toxicity relationship occurs prior to trial commencement and then Bayesian statistical modelling is used to incorporate accruing patient data during the trial to provide ongoing refinement of the dose–toxicity relationship),^52^ and designs that incorporate triggers based on disease responsiveness rather than just toxicity.^53^

While the likelihood of deriving therapeutic benefit from participation in Phase I cancer trials has historically been low, in the order of ~4%–5%,^54^ more recent analyses suggest that the balance of possible benefits to risks of Phase I trial participation has become more favourable over time. Illustrative evidence is provided by three studies which conducted retrospective evaluation of the rates of favourable response rates on imaging in Phase I cancer trials conducted via the National Cancer Institute's Cancer Therapy Evaluation Program (NCI‐CTEP) during the periods 1974–1982 (4.2%),^55^ 1991–2002 (10.6%)^56^ and 2000–2019 (12.2%),^57^ respectively. Notwithstanding some differences across study criteria (Supporting Information Material S1), these reports generally suggest an improvement in response rates over time, alongside reasonably stable, and low, rates of toxicity‐associated death around 0.49%^58^–0.7%.^59^ Suggestions to explain such increments include the emergence of more ‘rational’ drug development, of novel targeted therapeutics and immunotherapeutics compared to predominantly cytotoxic agents, as well as improvements in supportive care.^60^ Such data do not provide a full picture, however, given these studies fail to capture implications of participation in Phase I trials occurring beyond the US or pharmaceutical‐sponsored Phase I trials, and efficacy rates will inevitably vary between trials investigating differing therapeutic mechanisms and participant cohorts.

Various other authors have attempted to quantify the likelihood of benefiting from, or being harmed by, Phase I cancer trial participation; however, comparison between studies is challenged by the diversity of review methods as well as differing perspectives regarding appropriate metrics to reflect ‘benefit’ and ‘risk’ (e.g., some groups have examined tumour response rates on imaging,^61^ whereas others have focused on the frequency of receipt of ultimately approved agents,^62^ Supporting Information Material S1). The use of imaging response rates to capture benefit has been critiqued^63^ because this is generally considered to be a ‘surrogate’^64^ marker which may, or may not, translate to meaningful survival and/or quality‐of‐life improvements (or hold intrinsic value for patients^65^). The validity of other measures of response, such as disease stability, has also been debated. While, clinically, achievement of disease stability may well be advantageous for a patient if, for example, this delays the experience of disease‐related symptoms, the achievement of such benefits is difficult to quantify systematically.

The emergence of ‘precision medicine’ over the past two decades has also significantly altered the early‐phase clinical trials landscape. Increased understanding of the genomic and molecular drivers of disease has catalysed the development of targeted therapies which leverage specific molecular biomarkers. During drug development of targeted therapies, pre‐clinical understanding of a novel compound's molecular target may assist investigators in identifying the patient sub‐group most likely to benefit^66^ due to the presence of a specific biomarker. Several examples of targeted therapeutics for which response rates were notably favourable in their respective Phase I trials include crizotinib and osimertinib for the treatment of Non‐Small Cell Lung Cancer (NSCLC), in which objective response rates of 60.8% (95% confidence interval [CI]: 52.3%–68.9%)^67^ and 52% (95% CI: 30%–74%)^68^ were achieved respectively.

These examples do not on their own prove that trials of targeted therapies are more likely to benefit patients than trials of other types of agents. It is, however, important to recognise that the shift towards evaluation of targeted therapeutics and immunotherapeutics has also prompted and facilitated a number of other changes to Phase I trial design. Traditionally, Phase I cancer trials have been small (approximately 20–30 participants) and focused on dose‐finding, guided by safety, with efficacy subsequently evaluated in Phase II and then efficacy compared to standard‐of‐care in Phase III trials. More recently, there has been increasing use of large, multiple dose expansion cohort Phase I designs in which, subsequent to initial dose‐escalation (Phase Ia), additional participants may be recruited and further evaluation occurs in Phase Ib, such as in tumour or biomarker‐specific cohorts, or for further dose characterisation.^69^ In some instances, the ‘entire’ drug development pipeline can effectively be encompassed within a single multiple‐dose expansion cohort Phase I trial.^70^ Initial development of the immune checkpoint inhibitors, pembrolizumab, nivolumab and atezolizumab exemplify this trend.^71^ The incorporation of such design features complicates the quantification of participation benefits in modern Phase I trials, given likelihood of benefit will likely vary between Phase Ia and Ib. A systematic review and meta‐analysis of Phase I cancer trials evaluating novel targeted therapeutics conducted during 1 January 2015–1 July 2018 highlights this challenge, noting that objective response rates were higher among trials which included dose expansion cohorts (12.1%, 95% CI: 8.1–16.7 vs. 3.8%, 95% CI: 8.1–16.7, *p* < 0.01) and, separately, three‐fold higher in trials incorporating a biomarker eligibility criterion (*p* < 0.01).^72^

### 3.2 Recent changes to later‐phase trial designs

The nature of cancer clinical trial designs has also changed significantly in later‐phase settings. Two particular novel design features that depart from the standard two‐arm RCT model (in which the therapeutic misconception was initially noted) are the introduction of ‘master protocol’ trial designs and non‐fixed randomisation techniques.

‘Master protocol’ trials leverage one ‘common [trial protocol] infrastructure’^73^ however, in essence, consist of multiple sub‐studies, offering the means to explore utility of investigational agents across a variety of nuanced clinical scenarios, all encapsulated within the same over‐arching trial entity. Examples include ‘multi‐arm multi‐stage’ (MAMS) platform designs and ‘umbrella’ and ‘basket’ trials, with a notable increase in popularity of such designs over time, particularly within oncology^74^ (Table 1). The systemic therapy in advancing or metastatic prostate cancer: Evaluation of drug efficacy (STAMPEDE) trial offers one example of a MAMS platform trial.^75^ During the trial's recruitment period (2005–2023), over 10,000 men with locally advanced or metastatic prostate cancer were recruited, with multiple study comparator arms and a single control arm. As exemplified in STAMPEDE, as data emerge throughout the life of a MAMS study, additional, contemporaneous research questions can be incorporated, and study arms can be opened and closed as needed.^76^ Accordingly, while such trials have introduced new complexities, both with respect to statistical and practical issues throughout the trial design, planning and conduct phases,^77^ they have also been lauded for agility as well as efficiency compared to the process of conducting multiple, sequential two‐arm RCTs.^78^

**Table 1:** Examples of ‘master protocol’ trial designs.

Design	Description
Platform	Open‐ended trials, with capacity to add new investigational arms throughout the course of a trial (unlike standard RCTs), or cease under‐performing arms.
	Example: Systemic therapy in advancing or metastatic prostate cancer: Evaluation of drug efficacy (STAMPEDE) trial, phase III multi‐arm multi‐stage (MAMS) trial of treatments for men with locally advanced or metastatic prostate cancerr^a^
Umbrella	Participants with the same tumour type, with different investigational agents matched to different biomarkers
	Example: Biomarker‐integrated approaches of targeted therapy for lung cancer elimination (BATTLE) trial, phase II study, which enrolled participants with non‐small cell lung cancer^b^
Basket	Single investigational agent targeting specific biomarker, trial enrolls participants of varying tumour types with particular biomarker
	Example: *BRAF V600*, phase II, histology‐agnostic study evaluating the use of vemurafenib in participants with non‐melanomatous cancers harbouring *BRAF V600* alterations^c^

^a^ MRC Clinical Trials Unit, op. cit. note 75.

^b^ Kim, op. cit. note 79, pp. 44–53.

^c^ Hyman, D. M., Puzanov, I., Subbiah, V., Faris, J. E., Chau, I. Blay, J‐Y., Wolf, J., Raje, N. S., Diamond, E. L., Hollebecque, A., Gervais, R., Elez‐Fernandez, M. E., Italiano, A., Hofheinz, R.‐D., Hidalgo, M., Chan, E., Schuler, M., Lasserre, S. F., Makrutzki, M., Sirzen, F., Veronese, M. L., Tabernero, J., & Baselga, J. (2015). Vemurafenib in multiple nonmelanoma cancers with *BRAF V600* mutations. *The New England Journal of Medicine*, *373*(8), 726–736.

Novel randomisation methods, such as adaptive randomisation, offer a nuanced approach to subject allocation, leveraging data as it is generated throughout a trial. As such, adaptive randomisation aims to ‘optimise’ randomisation of trial participants to study arms that show early promise while also diverting participant allocation away from study arms which appear to be performing less favourably. This technique, which typically utilises Bayesian statistical techniques,^79^ has been increasingly adopted in cancer clinical trials. Adaptive randomisation has the advantage of being able to facilitate rapid testing of multiple study arms simultaneously, opening and closing study arms according to emerging signals within trial data, and potentially minimising costs (human, time and financial) of conducting multiple successive individual studies. For example, the biomarker‐integrated approaches of targeted therapy for lung cancer elimination (BATTLE) trial, a Phase II, randomised, open‐label study in people with advanced NSCLC,^80^ classified participants according to five molecular biomarker‐defined subgroups, with patients initially randomised in a routine fashion to 1–4 study intervention arms irrespective of biomarker status. Analysis was then undertaken at the 8‐week mark to determine whether there was any relationship between biomarker status and likelihood of tumour control between the different study arms. In this manner, determination of the ‘prior probability’ of tumour control for particular study interventions for participants with a certain biomarker could then be used to adaptively randomise subsequently enrolled participants. Ongoing refinement of the adaptive randomisation algorithm occurred throughout the study. Numerous clinical trials have subsequently employed adaptive randomisation techniques, capitalising on the advantage such techniques offer for simultaneous testing of multiple experimental interventions in order to identify a possible ‘winner’ for follow‐on confirmatory testing in large Phase III trials.^81^

There are some notable examples in which the evolution of trial designs appears to have enabled more efficient data generation and rapid translation into practice, as exemplified by clinical trials examining treatments for the management of *Human Epidermal growth factor Receptor 2* (*HER2*)‐amplified breast cancer. In studies of trastuzumab, the earliest anti‐*HER2* agent developed, 3,270 participants were recruited over 50 months, with 46 months of follow‐up on trial required to establish a lack of benefit in women without *HER2* amplification.^82^ In contrast, in the more recent I‐SPY2 trial, a Phase II adaptively randomised platform study, which investigated multiple therapeutic strategies for management of breast cancer, the average time for a drug ‘to graduate’ to a confirmatory phase III trial was 18 months.^83^ The results of this trial led to one of these drugs, pertuzumab, receiving accelerated approval by the FDA.^84^

While many master protocol designs, particularly umbrella and basket types, have been leveraged for the evaluation of ‘precision oncology’ approaches, there is mixed empirical data^85^ regarding whether such designs are any more likely than standard trial designs^86^ to be associated with higher likelihood of therapeutic benefit. For example, authors of a recent meta‐analysis of umbrella oncology trials published between 2006 and 2019 concluded that their ‘findings do not support the expectations of an increased benefit/risk ratio for participants of cancer umbrella trials’, although the authors noted that methodologic improvements over time of umbrella designs warrant reanalysis in future.^87^ It also merits noting that clinical trials employing ‘precision oncology’ approaches should not be conflated with ‘personalised’ oncologic care, given ‘they are not tailored to individual patients, but rather to subgroups of patients sharing only one of the several genetic alterations present in their tumours’.^88^ This is a nuanced concept to communicate to prospective trial participants during the consent process, however, and likely to become increasingly challenging with the emergence of other novel trial designs which further complicate this distinction (e.g., N‐of‐1 trial designs^89^).

## 4 IMPLICATIONS FOR THE THERAPEUTIC MISCONCEPTION

While methodological efficiencies are desirable, the increased integration of research into practice along with the emergence of novel trial designs have major implications for the therapeutic misconception. Notably, the changes outlined are likely to simultaneously increase the likelihood of the therapeutic misconception occurring while at the same time, paradoxically, increasing the likelihood of benefit from trial participation—thereby reducing the clinical and moral salience of the therapeutic misconception. As such, it is also important to recognise that expectations (of participants and/or clinicians) of benefit from research in the modern clinical trials era may not always be a misconception (Table 2).

**Table 2:** Implications of recent changes to the clinical trials landscape for understanding among research participants.

	Factor	Possible implication/s
Factors which may increase the likelihood of the therapeutic misconception and/or related misunderstandings regarding research by trial participants	Decentralisation of clinical trials away from dedicated academic research centres (e.g., increased uptake of tele‐trials during the COVID‐19 pandemic and beyond)	Routine clinical care and clinical trial participation more likely to occur at the same centre, conducted by the same clinicians (i.e., may make the care‐research distinction less obvious for patients).
	Increasing complexity of trial designs and divergence away from simple two‐arm RCTs (e.g., MAMS designs, adaptive randomisation)	Task of comprehension is made harder, potential to further disadvantage already under‐represented groups in clinical trials (e.g., patients with lower educational status, non‐English speaking background). Participant understanding of subject allocation in Bayesian adaptive randomisation procedures remains unexamined. Noting empirical evidence of misunderstandings regarding standard ‘by chance’ randomisation, there is the risk of further misunderstandings occurring, including the impression that adaptive randomisation is principally designed to serve the best interests of individuals.^a^
	Individuals function as clinicians as well as trial investigators/scientists	Potential conflict between traditional clinician duties for ‘personalised care’ versus investigator responsibility of fidelity towards study protocol (the ‘dual agency’ dilemma). It may be difficult for research participants to appreciate the different degree of uncertainty in individuals conveying information regarding potential risks and benefits of standard care treatment (as clinicians) versus in a clinical trial context (as researchers)^b^
Factors which may make clinical research more therapeutic, and hence diminish concerns regarding the therapeutic misconception or therapeutic orientation towards clinical trials	Increased response rates in modern compared to historical phase I trials (in oncology, this is largely because understanding of the molecular basis of cancer and drug targets has been advanced, allowing more rational drug development)	Patients can correctly assume that they might benefit from trials. They might also expect more rapid dose escalation and selection according to biomarker status. In this context, the challenge is to explain to patients that responses will still be heterogeneous (influenced by patient and intervention factors) to avoid overestimation of likelihood of benefit. While traditionally participants enrolled in phase I trials were patients with end‐stage disease with no remaining standard‐of‐care options, this may no longer always be the case. For example, for patients recognised to have a particular targetable biomarker, consideration of enrolment in phase I trials of targeted therapeutics may occur even if there are other, non‐targeted, standard options remaining.
	Movement towards ‘learning health systems’ in which research and care are integrated	Benefits of research are more readily integrated within routine care, so patients would be correct in assuming that there is overlap between research and care.
	Use of cross‐over design features in later‐phase, randomised trial designs. Patients randomised to standard‐of‐care arms can receive access to the interventional arm therapeutic at the point of disease progression	Patients can rightly expect to receive access to the experimental intervention at some point in a study, which increases their likelihood of benefiting from participation.
	Inclusion of complimentary molecular testing platforms as part of pre‐screening in some clinical trials to identify patients with the relevant biomarker for inclusion in the trial	May facilitate identification of other biomarkers which can be leveraged therapeutically in a patient's care (whether via a clinical trial or routine care). Advantageous for patients who would not otherwise be able to access commercially available molecular testing platforms due to cost.

^a^ Hey, op. cit. note 116, pp. 102–106.

^b^ Kim, S. Y. H., De Vries, R., Parnami, S., Wilson, R., Kim, H.M., Frank, S., Holloway, R. G., & Kieburtz, K. (2015). Are therapeutic motivation and having one's own doctor as researcher sources of therapeutic misconception? *Journal of Medical Ethics*. *41*(5), 391–397.

### 4.1 Increased risk of the therapeutic misconception

As modern clinical trials diverge further away from straightforward two‐arm RCT designs, the task of comprehension for participants is made harder and the risk of the therapeutic misconception is likely exacerbated. The extent to which it occurs in trials that utilise adaptive randomisation or MAMS platforms has yet to be examined—however, there is good reason to expect that participants will have considerable difficulty distinguishing such trials from clinical practice and/or understanding their likelihood of benefit. Trials that engage Bayesian statistical techniques^90^ rely on complex and ongoing statistical modelling. It is unclear how participants navigate such complexity, or for that matter how clinicians and researchers understand them and convey them to trial participants. Problems in statistical interpretation and understanding of commonly reported trial endpoints have previously been demonstrated among clinicians.^91^ Further study (including outside North America) will be required to map the prevalence of the therapeutic misconception in the context of trials that use novel randomisation techniques. In this regard, it is pertinent to note that empirical studies to date of the therapeutic misconception and participant understanding have been largely conducted among highly educated trial participants at academic institutions^92^ or dedicated clinical trial centres.^93^ If even these groups have difficulty comprehending the distinctions between therapy and research, then it is highly likely that the therapeutic misconception will be intensified as trial designs evolve.

Practical factors, such as the ‘where’ and by ‘whom’ modern clinical trial conduct occurs, are also likely to contribute to the therapeutic misconception among research participants. The growing trend for decentralisation of clinical trial conduct away from dedicated clinical trial centres, including the uptake of tele‐trials models buoyed by the COVID‐19 pandemic,^94^ has been beneficial for many patients with respect to trial access, particularly patients based in rural settings.^95^ However, when patients, increasingly, no longer travel away from the site at which they receive routine care in order to access trial opportunities and, additionally, may receive both routine care and clinical trial management from the same individual (i.e., the clinician‐researcher), it may be harder to separate one type of care from the other.

### 4.2 Reduced salience of the therapeutic misconception

Somewhat paradoxically, at the same time as changes to the clinical trials landscape may enhance the likelihood of the therapeutic misconception occurring, changes may also validate, at least in part, belief held by some participants (and clinicians, researchers and policy orientations) that research has a therapeutic intent.^96^ While it is already well‐recognised that participants may benefit from trial participation due to enhanced monitoring, the placebo effect and luck (if allocated to an experimental intervention that proves to be effective), it is important to now also consider a further possibility: that the threshold of biological plausibility may now be higher, and the dynamism of trial design has been increased, and so patients’ expectations regarding benefit from trial participation may, potentially, hold greater weight. Even Phase 1 studies, which are classically not designed to prove efficacy, may increasingly have a therapeutic orientation and be offered to patients earlier on in their disease trajectory than has previously been the case.^97^

It may, therefore, increasingly be the case that it is neither an error to see research as a type of clinical practice nor to anticipate benefit. Of course, the prospect of therapeutic benefit will still vary considerably between trials according to phase (I–III), disease, and therapeutic mechanism (e.g., cytotoxics, targeted therapeutics, immunotherapeutic, and combination approaches), and, even within therapeutically oriented trials, every participant could not expect to benefit.^98^ Nevertheless, patients considering participation in modern clinical trials, including early‐phase designs, might now be affirmed, at least to some extent, in expectations of benefit from trial participation, particularly in the context of biomarker‐matched trials of novel targeted therapeutics.^99^

## 5 IMPLICATIONS FOR RESEARCH ETHICS

Both the increased likelihood of the therapeutic misconception and potential reductions in its salience have important implications for research ethics, including for trial design, trial access and participant selection, communication and consent, and role delineation.

### 5.1 Trial design

It has long been a tenet of research ethics that it is only ethical to randomise participants in trials if there is genuine uncertainty (at least in the scientific community at large) as to whether the experimental intervention offers any benefit over the comparator (placebo or standard of care).^100^ This state of uncertainty is often referred to as collective ‘equipoise’. If it is the case that trials are becoming more therapeutic in their orientation, the idea that ‘equipoise’ is a necessary prerequisite is also increasingly challenged. For example, in the adaptively randomised phase II I‐SPY2 trial, novel investigational agents were flagged for ‘graduation’ to a confirmatory phase III RCT following achievement of a >85% ‘estimated probability of success’ threshold.^101^ While apparent promise in a phase II trial does not necessarily predict significant efficacy in a phase III RCT, nor indeed effectiveness in the ‘real‐world’ post‐trial setting,^102^ new trial designs nonetheless prompt questions about both whether equipoise exists and whether it is a necessary condition of ethical trial design. Some commentators have also suggested that adaptive randomisation risks premature disruption of equipoise as data are accrued and reviewed by investigator teams throughout a trial,^103^ whereas others, such as Alex London, argue that collective conceptualisations of equipoise remain appropriately preserved given uncertainty regarding best practice still exists beyond the investigator team.^104^

Another methodological implication of trials becoming more therapeutically oriented is that there will be a greater ethical imperative to design trials that seek to maximise benefits for participating patients in addition to generating knowledge for the benefit of future patients,^105^ for example, trials that allow patients to ‘cross over’ from one treatment arm to another, when appropriate,^106^ and to follow up blinded, comparative studies with ‘open label’ extensions, such that all patient‐participants in clinical trials have equal opportunities to benefit. Simultaneously, such design features, at times, may challenge the ability to identify statistically significant differences between study arms (e.g., if there is significant cross‐over and the difference in effect size is modest between interventions), meaning that it will be critically important to iteratively address the tensions that may arise between ethics and epistemology in clinical research.

### 5.2 Access and participant selection

As trials become more therapeutic, equity of access to clinical trials and issues of participant diversity and inclusion will become increasingly morally salient.^107^ The need to give attention towards patient groups already under‐represented in clinical trials, such as patients of non‐English speaking backgrounds, patients living in rural areas, or those of lower socioeconomic status, will be further underlined.^108^ Moreover, while trial eligibility criteria are, traditionally, strict, there may be an increased imperative to ensure that eligibility criteria are such that trial populations more readily reflect real‐world populations (e.g., patients with other comorbidities).^109^ In this regard, as clinical trials become a means of accessing novel therapeutics and diagnostic platforms, they may either redress or deepen inequities of access depending on how accessible and inclusive trials and study protocols are.

There may also be a greater ethical imperative for trials to be integrated into practice enabling greater patient access; for all sites to offer access to trials (whether on‐site, tele‐trials, or via onward referral to other sites) and for more clinicians to become clinician‐scientists.^110^ Furthermore, it may paradoxically be the case going forward that if a clinician elects *not* to refer an eligible patient for available clinical trials, there exists a moral impetus for justification as to why this is the case.^111^

More broadly, there may be more of a need for regulators to consider approving interventions on the basis of earlier phase studies (as is already occurring through a wide variety of ‘accelerated access’ programmes internationally). This may be driven by the need for trial designs to manage increasingly small, biomarker‐defined, patient cohorts which challenge the feasibility (and timeliness) of conducting large‐scale RCTs, as well as consumer advocacy for efficient access to novel therapeutics (particularly so in the context of life‐limiting illnesses such as cancer).^112^ However, it is important that political, commercial and consumer pressure for such programmes does not erode clinical trial standards, as even drugs that have biological plausibility and appear promising in earlier phase trials are not always found to have benefit in Phase III RCTs^113^ (or benefit in the post‐RCT ‘real world’ setting). Likewise, regulatory mechanisms to promote ongoing data collection will be crucial (regarding real‐world ‘effectiveness’ as well as long‐term safety and tolerability data), particularly for drugs that have received ‘accelerated approval’. In this regard, it is noteworthy that a retrospective audit of cancer drugs granted accelerated approval by the FDA up until December 2020 showed that the benefits of 10 anti‐cancer agents, encompassing 18 different treatment indications, could not be confirmed, either because subsequent trials did not corroborate the results of earlier trials, or such studies were not conducted.^114^ Of some concern, at the time of publication, ongoing endorsement of some agents was identified in national clinical guidelines.^115^

### 5.3 Communication and consent

Both increased complexity of trials and their increasing therapeutic orientation have major implications for communication and participant consent. Here, great care needs to be taken to address both the increased likelihood of the therapeutic misconception and/or to communicate to patients that they may, in fact, reasonably expect to benefit from research participation. With respect to the former (ongoing and exacerbated therapeutic misconception), it will be necessary to devise new ways of informing—and keeping people informed—about the nature of complex study designs and how they *differ* from clinical care. These differences may be subtle—for example, an adaptive trial differs from clinical care in the sense that decisions about trial redesign are made for groups, rather than for individuals. With respect to the latter, it will be necessary to explain how trials are *similar* to clinical care and what kinds of benefits participants can reasonably expect, while ensuring that these expectations do not become the basis of undue inducement. It is also important that participants are aware that trial participation—even if it is ‘therapeutic’—entails monitoring that might not ordinarily be done as part of routine care. This might not only be inconvenient but could also carry risk for the research participant—for example, a procedural complication from a tissue biopsy.

Specific emerging trial designs might require particular modifications to consent processes. For example, where there is adaptive randomisation, questions arise as to whether patients randomised earlier on in a trial's conduct require different information during the informed consent process to those randomised later on (when more ‘learning’ has been done by the adaptive randomisation algorithm). On the one hand, such trials are designed *a priori* to flexibly integrate accruing data, and it would be expected that participants who agree to participate are aware of this at enrolment. However, whether or not participants understand and appreciate the implications of enrolling at different points in a study is less clear.

Adaptive randomisation also raises questions about whether ongoing (re)consent is required. Standard research ethics principles mandate ‘new information’ arising during a trial's conduct be relayed to participants, on the basis of respect for autonomy.^116^ Whether, and if so when, the evolution of the randomisation procedure meets the threshold for ‘new’ information is a matter of ongoing debate. These issues become particularly salient in the targeted therapy era, in which evolving data may redirect participants with particular biomarkers away from certain treatment allocations later on in a study's conduct (e.g., the BATTLE trial described above). Ethicists Hey and Kimmelman suggest that there may be a greater need to manage risk of the therapeutic misestimation in trials incorporating adaptive randomisation.^117^ They also highlight that while adaptive randomisation preferences subject allocation towards more favourably performing study arms, some participants will continue to be allocated to the less well‐performing arm, and that this should be made explicit to prospective participants, albeit they also acknowledge this could be challenging for participants to understand and, also, impact recruitment.^118^

‘Dynamic consent’ platforms,^119^ which facilitate communication of information to trial participants at different points in time, may be useful for trials involving adaptive randomisation or those that have MAMS features. One example, the ‘CTRL’ online platform, was designed to facilitate consent for participants on a molecular profiling study, and has demonstrated several advantages to traditional ‘once‐off’ consent, including: contained information disclosure via smaller ‘chunks’ over a period of time to avoid participants being overwhelmed with information, opportunity for participants to consent to the breadth of a study's research portfolio or particular research questions only, and the opportunity to invite participants to have their data utilised for new research questions which have arisen subsequent to initial enrolment.^120^ Such features offer advantages for participants, by promoting ‘ongoing participant‐led management of their involvement in research studies’, as well as for researchers, by creating mechanisms to update consent for salient research questions which evolve over the life of the study.^121^ Further research would be required to conceptualise a dynamic consent interface for an interventional rather than observational study like CTRL, as well as evaluate practical aspects such as feasibility and acceptability in the real‐world setting among various stakeholders (i.e., consumers, researchers and ethics committees).

To date, efforts to develop and test strategies for mitigating the risk of the therapeutic misconception have been limited. In one study by Christopher et al., participants with a variety of conditions were randomised to routine informed consent versus delivery of an educational video outlining aspects of the hypothetical clinical trial, with the intervention group exhibiting less therapeutic misconception.^122^ Further research, across a range of participant demographics, and involving participants enrolling in real (rather than hypothetical) trials, of varying phases and designs and with varying likelihood of benefit, will be required to incorporate both increasing trial complexity and the increased therapeutic orientation of some trials.

### 5.4 Role delineation

In many codes of research ethics, it is argued that there should usually be a separation between the role of the researcher and that of the clinician whose primary concern is the ‘best interests’ of the participant rather than knowledge generation.^123^ This role delineation is believed to be important at all stages of research, including recruitment, selection, consent, monitoring, reporting of adverse events, outcome assessment and decisions about continuation, withdrawal and trial cessation. Another argument for separating these roles is that clinicians recruiting patients will create or exacerbate a therapeutic misconception.

This role delineation has always been difficult and contested—for example, some patients have a preference for having clinicians rather than researchers recruit them to trials, and some might argue that it should be the clinician who has longitudinal familiarity with a patient's medical history, values and preferences, as well as available clinical trials and standard‐of‐care options, who is best placed to conduct the informed consent process. Furthermore, there is value to be gained by promoting research‐oriented clinicians who, through their clinical practice, identify clinically meaningful research questions that form the backbone of ‘investigator‐initiated’ clinical trials. In addition, good medical care should be informed by evidence and support ongoing evidence generation.^124^ As Kimmelman has argued, adopting a siloed relationship between research and care may not only be naïve but also problematic, and plurality in trial (and investigator) objectives (to advance science and to benefit research participants) should be encouraged.^125^

The assumption that clinical and research roles should be sharply delineated becomes increasingly problematic where trials are routinely integrated into clinical practice and where there are genuine possibilities that trial participants will benefit from research (either by being more closely monitored or by receiving an agent with therapeutic benefit), or at least that it offers the ‘best’ option for them. Specifically with respect to the therapeutic misconception, greater involvement of clinicians in research might further entrench such misconceptions. At the same time, as trials become more ‘therapeutic’, it may become less problematic for patients to see overlaps between care (and carers) and research (and researchers). Of course, it remains important for clinician‐investigators to explain the nuances of their roles to patients and disclose significant potential conflicts of interest during the consent process. This may include, for example, financial interests in the agent under investigation or trial sponsor entity, as well as intellectual or professional interests (e.g., when the clinician may stand to benefit professionally from timely recruitment or completion or publication of the study).

### 5.5 Broader implications

Notwithstanding the possible benefits of novel trial designs discussed, it is crucial that moves to nuance clinical care and communication are not used as a rhetorical device to obscure the real uncertainties and risks associated with investigational drugs given the broader epistemic goals of research with respect to evidence generation. This is particularly important given the push by industry, governments and consumer groups^126^ to register and subsidise therapies on the basis of ever‐decreasing levels of evidence (e.g., through accelerated access and provisional approval processes). We emphasise that, in arguing that we should update the way we communicate with patients about trial participation, we are not arguing that evidence standards should be eroded in regulatory and funding decisions.

## 6 CONCLUSION

Clinicians, researchers and ethicists have long had an interest in why patients participate in clinical trials. A persisting concern is that patients are motivated, in part, by misconceptions and misunderstandings of trial designs and their implications, and the likelihood of benefit. It is increasingly likely that these concerns will need rethinking as clinical care and research become more entwined, trial designs become more rational and tailored to patients' biological characteristics, and trial designs become more directed towards providing benefit to trial participants (in addition to generating knowledge to benefit future patients). Further philosophical and ethical reflection about research ethics and about research more broadly, will be important. Changes in the ways that trials are designed, conducted and integrated make it imperative that such reflections are informed by all those who have a stake in research, including government, research sponsors, clinicians, patients and consumer advocates.

## CONFLICTS OF INTEREST STATEMENT

Tom John: Honoraria/Advisory: BMS, AstraZeneca, Amgen, Roche, Pfizer, Takeda, Boehringer Ingelheim, MSD, Merck, Puma, Specialised Therapeutics, Gilead, Seagen, Gilead. Travel/speaker fees: AstraZeneca, MSD. Sue‐Anne McLachlan: Advisory Board: BMS, Educational activities: BMS. Sarah Heynemann, Wendy Lipworth, Jennifer Philip, and Ian Kerridge declare no conflict of interest.

## Supporting information

Supporting information.

## Footnotes

1 Appelbaum, P. S., Roth, L. H., & Lidz, C. (1982). The therapeutic misconception: Informed consent in psychiatric research. *International Journal of Law and Psychiatry*, *5*(3‐4), 319–329.

2 Ibid: 327.

3 Miller, F. G., & Rosenstein, D. L. (2003). The therapeutic orientation to clinical trials. *The New England Journal of Medicine*, *348*(14), 1383–1386.

4 Miller, F. G., & Brody, H. (2003). A critique of clinical equipoise: Therapeutic misconception in the ethics of clinical trials. *The Hastings Center Report*, 33(3), 19–28.

5 Appelbaum, P. S., et al., op. cit. note 1, p. 321.

6 Joffe, S., & Miller, F. G. (2008). Bench to bedside: mapping the moral terrain of clinical research. *The Hastings Center Report*, *38*(2), 30–42; National Commission for the Protection of Human Subjects of, Biomedical Behavioral Research, Bethesda M. D. (1978)*. The belmont report*. https://nla.gov.au/nla.cat-vn5379101

7 Abernethy, E. R., Campbell, G. P., Hianik, R. S., Thomson, M. C., Blee, S. M., Sibold, H. C., Dixon M. D., Switchenko, J. M., & Pentz, R. D. (2021). Reassessing the measurement and presence of therapeutic misconception in a phase 1 setting. *Cancer*, *127*(20), 3794–3800; Shumway, D. A., Motomura, A., Griffith, K. A., Hayman, J. A., Pierce, L. J., & Jagsi, R. (2020). Patient perceptions in a nonblinded randomized trial of radiation therapy technologies: A novel survey study exploring therapeutic misconception. *International Journal Radiation Oncology Biology Physics*, *108*(4), 867–875; Malan, T., & Moodley, K. (2016). Phase 3 oncology clinical trials in South Africa: Experimentation or therapeutic misconception? *Journal Empirical Research Human Research Ethics*, *11*(1), 47–56; Escritt, K., Mann, M., Nelson, A., & Harrop, E. (2022). Hope and meaning‐making in phase 1 oncology trials: A systematic review and thematic synthesis of qualitative evidence on patient‐participant experiences. *Trials*, *23*(409), 1–15.

8 Abernethy, op. cit. note 7, pp. 3794–3800; Escritt, op. cit. note 7, pp. 1–15; Pentz, R. D., White, M., Harvey, R. D., Farmer, Z. L., Liu, Y., Lewis, C., Dashevskaya, O., Owonikoko, T., & Khuri, F. R. (2012). Therapeutic misconception, misestimation, and optimism in participants enrolled in phase 1 trials. *Cancer*, *118*(18), 4571–4578.

9 Abernethy, op. cit. note 7, pp. 3794–3800; Shumway, op. cit. note 7, pp. 867–875; Reeder‐Hayes, K. E., Roberts, M. C., Henderson, G. E., & Dees, E. C. (2017). Informed consent and decision making among participants in novel‐design phase I oncology trials. *Journal Oncology Practice*, *13*(10), e863–e873.

10 Shumway, op. cit. note 7, pp. 867–875; Godskesen, T., Nygren, P., Nordin, K., Hansson, M., & Kihlbom, U., (2013). Phase 1 clinical trials in end‐stage cancer: Patient understanding of trial premises and motives for participation. *Supportive Care Cancer*, *21*(11), 3137–3142.

11 Engelbak Nielsen, Z., Eriksson, S., Schram Harsløf, L. B., Petri, S., Helgesson, G., Mangset, M., & Godskesen, T. E. (2020). Are cancer patients better off if they participate in clinical trials? A mixed methods study. *BMC Cancer*, *20*(401), 1–17.

12 Lai‐Kwon, J., Heynemann, S., Flore, J., Dhillon, H., Duffy, M., Burke, J., Briggs, L., Leigh, L., Mileshkin, L., Solomon, B., Ball, B., Kokanovic, R., & Jefford, M. (2021). Living with and beyond metastatic non‐small cell lung cancer: the survivorship experience for people treated with immunotherapy or targeted therapy. *Journal Cancer Survivorship*, *15*(3), 392–397.

13 Bui, T., Liang, R., Kiely, B. E., Brown, C., Dhillon, H. M., & Blinman, P. (2021). Scanxiety: A scoping review about scan‐associated anxiety. *BMJ Open*, *11*(5), e043215, 1–22.

14 Ibid.

15 Gupta, A., Eisenhauer, E. A., & Booth, C. M. (2022). The time toxicity of cancer treatment. *Journal of Clinical Oncology*, *40*(15), 1611–1615.

16 Miller & Rosenstein, op. cit. note 3, pp. 1383–1386.

17 Green, J. M. (2012). Therapeutic misdirection: An analysis of protocol exception requests in clinical trials. *Journal of Empirical Research on Human Research Ethics*, *7*(5), 37–43.

18 Ibid: 38.

19 Appelbaum, op. cit. note 1, pp. 319–329.

20 Horng, S., & Grady, C. (2003). Misunderstandings in clinical research: Distinguishing therapeutic misconception, Therapeutic misestimation, & therapeutic optimism. *IRB: Ethics & Human Research*, *25*(1), 11–16.

21 Ibid.

22 Sisk, B. A., & Kodish, E. (2018). Therapeutic misperceptions in early‐phase cancer trials: From categorical to continuous. *IRB: Ethics and Human Research*, *40*(4), 13–20.

23 Jansen, L. A. (2016). The optimistic bias and illusions of control in clinical research. *IRB: Ethics and Human Research*, *38*(2), 8–14; Jansen, L. A., Mahadevan, D., Appelbaum, P. S., Klein, W. M., Weinstein, N. D., Mori, M., Daffé, R., & Sulmasy, D. P. (2016). Dispositional optimism and therapeutic expectations in early‐phase oncology trials. *Cancer*, *122*(8), 1238–1246; Jansen, L. A., Mahadevan, D., Appelbaum, P. S., Klein, W. M. P., Weinstein, N. D., Mori, M., Degnin, C., & Sulmasy, D. P. (2017). Variations in unrealistic optimism between acceptors and decliners of early phase cancer trials. *Journal Empirical Research Human Research Ethics*, *12*(4), 280–288.

24 Kimmelman, J. (2007). The therapeutic misconception at 25: Treatment, research, and confusion. *The Hastings Center Report*, *37*(6), 36–42.

25 Jansen (2016), op. cit. note 23, pp. 8–14; Jansen, L. A., et al. (2016), op. cit. note 23, pp. 1238–1246; Jansen, L.A., et al. (2017), op. cit. note 23, pp. 280–288.

26 Horng & Grady, op. cit. note 20, pp. 11–16.

27 Appelbaum, P. S., et al., op. cit. note 1, pp. 319–329; Appelbaum, P. S., & Lidz, C., (2008). The therapeutic misconception. In Emanuel, E. J., et al, *The Oxford handbook of clinical research ethics*. Oxford University Press.

28 Abernethy, op. cit. note 8, pp. 3794–3800; Malan, op. cit. note 7, pp. 47–56; Pentz, op. cit. note 8, pp. 4571–4578; Campbell, G., Dixon, M. D., Lohani, M., Cook, J., Hianik, R., Thomson, M. C., Abernethy, E., Lewis, C., Switchenko, J., Harvey, R. D., & Pentz, R. D. (2022). Therapeutic misconception about research procedures: Does a simple information chart improve understanding? *Ethics Human Research*, *44*(22), 18–25; Gota, V., Nookala, M., Yadav, A., Menezes, S. R., Kannan, S., & Ali, R. (2018). Quality of informed consent in cancer clinical trials in India: A cross‐sectional survey. *National Medical Journal of India*, *31*(6), 334–338.

29 Abernethy, op. cit. note 8, pp. 3794–3800; Pentz, op. cit. note 8, pp. 4571–4578.

30 Abernethy, op. cit. note 8, pp. 3794–3800; Weinfurt, K. P., Sulmasy, D. P., Schulman, K. A., & Meropol, N. J. (2003). Patient expectations of benefit from phase I clinical trials: Linguistic considerations in diagnosing a therapeutic misconception. *Theoretical Medicine and Bioethics*, *24*(4), 329–344.

31 Abernethy, op. cit. note 8, pp. 3794–3800; Jansen (2016), op. cit. note 23, pp. 1238–1246; Christopher, P. P., Appelbaum, P. S., Truong, D., Albert, K., Maranda, L., & Lidz, C. (2017). Reducing therapeutic misconception: Randomized intervention trial in hypothetical clinical trials. *PLoS One*, *12*(9), e0184224, 1–11.

32 Jansen (2016), op. cit. note 23, pp. 1238–1246.

33 Appelbaum, P. S. (2007). Assessment of patients' competence to consent to treatment. *The New England Journal of Medicine*, *357*(18), 1834–1840.

34 Sisk, op. cit. note 22, pp. 13–20.

35 Henderson, G. E., Churchill, L. R., Davis, A. M., Easter, M. M., Grady, C., Joffe, S., Kass, N., King, N. M. P., Lidz, C. W., Miller, F. G., Nelson, D. K., Peppercorn, J., Rothschild, B. B., Sankar, P., Wilfond, B. S., & Zimmer, C. R. (2007). Clinical trials and medical care: Defining the therapeutic misconception. *PLoS Medicine*, *4*(11), e324, 1735–1738.

36 National Commission for the Protection of Human Subjects of Biomedical Behavioral Research, Bethesda M. D., op. cit. note 6.

37 National Comprehensive Cancer Network®. (2022). *NCCN clinical practice guidelines in oncology (NCCN Guidelines*®)*: Breast cancer, Version 4*. https://www.nccn.org/professionals/physician_gls/pdf/breast.pdf

38 Weber, J. S., Levit, L. A., Adamson, P. C., Bruinooge, S., Burris, H. A., Carducci, M. A., Dicker, A. P., Gönen, M., Keefe, S. M., Postow, M. A., Thompson, M. A., Waterhouse, D. M., Weiner, S. L., & Schuchter, L. M. (2015). American Society of Clinical Oncology Policy Statement Update: The critical role of phase I trials in cancer research and treatment. *Journal of Clinical Oncology*, *33*(3), 278–284.

39 Peter MacCallum Cancer Centre. (2002). *Clinical trial care*. https://www.petermac.org/clinical-trial-care

40 Levit, L., Balogh, E., Nass, S., & Ganz, P. A. (2013). *Delivering high‐quality cancer care: charting a new course for a system in crisis*. National Academies Press.

41 Smith, M., Saunders, R., Stuckhardt, L., & McGinnis, J. M. (2013). *Best care at lower cost: The path to continuously learning health care in America*. National Academies Press.

42 Agency for Healthcare Research and Quality. (2019). *About learning health systems*. https://www.ahrq.gov/learning-health-systems/about.html

43 Faden, R. R., Kass, N. E., Goodman, S. N., Pronovost, P., Tunis, S, & Beauchamp, T. L. (2013). An ethics framework for a learning health care system: A departure from traditional research ethics and clinical ethics. *The Hastings Center Report*, *43*(s1), S16–S27.

44 Zhang, S. X., Fergusson, D., & Kimmelamn, J. (2020). Proportion of patients in phase I oncology trials receiving treatments that are ultimately approved. *Journal of the National Cancer Institute*, *112*(9), 886–892.

45 Chihara, D., Lin, R., Flowers, C. R., Finnigan, S. R., Cordes, L. M., Fukuda, Y., Huang, E. P., Rubinstein, L. V., Nastoupil, L. J., Ivy, S. P., Doroshow, J. H., & Takebe, N. (2022). Early drug development in solid tumours: Analysis of National Cancer Institute‐sponsored phase I trials. *The Lancet*, *400*(10351), 512–521.

46 Weber, op. cit. note 38, p. 281.

47 Ibid: 278–284.

48 Kimmelman, J. (2019). Phase I trials as therapeutic options: (Usually) a betrayal of evidence‐based medicine. *Nature Reviews Clinical Oncology*, *16*(12), 719–721; Kimmelman, J. (2020). Reply to ‘correcting the ASCO position on phase I clinical trials in cancer’. *Nature Reviews Clinical Oncology*, *17*(2), 125–126.

49 Burris, H. A. (2020). Correcting the ASCO position on phase I clinical trials in cancer. *Nature Reviews Clincal Oncology*, *17*(2), 125.

50 Hey, S. P., & Kimmelman, J. (2014). The risk‐escalation model: A principled design strategy for early‐phase trials. *Kennedy Institute of Ethics Journal*, *24*(2), 121–139.

51 Kurzrock, R., Chia‐Chi, L., Tsung‐Che, W., Hobbs, B., Carmagnani, R., Pestana, M.D., & Hong, D. S. (2021). Moving beyond 3 + 3: The future of clinical trial design*. American Society of Clinical Oncology Educational Book*, *41*, e133–e144.

52 Weber, op. cit. note 38, pp. 278–284; Hansen, A. R., Graham, D. M., Pond, G. R., & Siu, L. L. (2014). Phase 1 trial design: Is 3 + 3 the best? *Cancer Control*, *21*(3), 200–208.

53 Weber, op. cit. note 38, pp. 278–284; Rogatko, A., Cook‐Wiens, G., Tighiouart, M., & Piantadosi, S. (2015). Escalation with overdose control is more efficient and safer than accelerated titration for dose finding. *Entropy*, *17*(8), 5288–5303.

54 Weber, op. cit. note 38, pp. 278–284; Estey, E., Hoth, D., Simon, R., Marsoni, S., Leyland‐Jones, B., & Wittes, R. (1986). Therapeutic response in phase I trials of antineoplastic agents. *Cancer Treatment Reports*, *70*(9), 1105–1115.

55 Estey, op. cit. note 54, pp. 1105–1115.

56 Horstmann, E., McCabe, M.S., Grochow, L., Yamamoto, S., Rubinstein, L., Budd, T., Shoemaker, D., Emanuel, E. J., & Grady, C. (2005). Risks and benefits of phase 1 oncology trials, 1991 through 2002. *The New England Journal of Medicine*, *352*(9), 895–904.

57 Chihara, op. cit. note 45, pp. 512–521.

58 Horstmann, op. cit. note 56, pp. 895–904.

59 Chihara, op. cit. note 45, pp. 512–521.

60 Weber, op. cit. note 38, pp. 278–284; Ibid.; Mackley, M. P., Fernandez, N. R., Fletcher, B., Woolcott, C. G., & Fernandez, C. V. (2021). Revisiting risk and benefit in early oncology trials in the era of precision medicine: A systematic review and meta‐analysis of phase I trials of targeted single‐agent anticancer therapies. *Journal of Clinical Oncology—Precision Oncology*, *2021*(5), 17–26.

61 Chihara, op. cit. note 45, pp. 512–521; Horstmann, op. cit. note 56, pp. 895–904; Mackley, op. cit. note 60, pp. 17–26; Roberts, T. G., Goulart, B. H., Squitieri, L., Stallings, S. C., Halpern, E. F., Chabner, B. A., Gazelle, G. S., Finkelstein, S. N., & Clark, J. W. (2004). Trends in the risks and benefits to patients with cancer participating in phase 1 clinical trials. *Journal of the American Medical Association*, *292*(17), 2130–2140; Italiano, A., Massard, C., Bahleda, R., Vataire, A. L., Deutsch, E., Magné, N., Pignon, J. P., Vassal, G., Armand, J. P., & Soria, J. C. (2008). Treatment outcome and survival in participants of phase I oncology trials carried out from 2003 to 2006 at Institut Gustave Roussy. *Annals Oncology*, *19*(4), 787–792.

62 Zhang, op. cit. note 44, pp. 886–892.

63 Ibid; Kimmelman (2019), op. cit. note 48, pp. 719–721.

64 Bruzzi, P., Del Mastro, L., Sormani, M. P., Bastholt, L., Danova, M., Focan, C., Fountzilas, G., Paul, J., Rosso, R., & Venturini, M. (2005). Objective response to chemotherapy as a potential surrogate end point of survival in metastatic breast cancer patients. *Journal of Clinical Oncology*, *23*(22), 5117–5125.

65 Robinson, A. G., O'Donnell, J., Booth, C. M., Koven, R., Eisenhauer, E. A., & Brundage, M. D. (2021). Patient perspectives of value of delayed disease progression on imaging (imaging PFS). A treatment trade‐off experiment. *Journal of Cancer Policy*, *30*(100301); Brundage, M. D., Booth, C. M., Eisenhauer, E. A., Galica, J., Kankesan, J., Karim, S., Koven, R., McDonald, V., Ng, T., O'Donnell, J., tenHove, J., Robinson, A. (2023). Patients' attitudes and preferences toward delayed disease progression in the absence of improved survival. *Journal of the National Cancer Institute* 115(12):1526–1534. https://doi.org/10.1093/jnci/djad138

66 Weber, op. cit. note 38, pp. 278–284; Chihara, op. cit. note 45, pp. 512–521.

67 Camidge, D. R., Bang, Y. J., Kwak, E. L., Iafrate, A. J., Varella‐Garcia, M., Fox. S. B., Riely, G. J., Solomon, B., Ou, S‐H. I., Kim, D‐W., Salgia, R., Fidias, P., Engelman, J. A., Gandhi, L., Jänne, P. A., Costa, D. B., Shapiro, G. I., Lorusso, P., Ruffner, K., Stephenson, P., Tang, Y., Wilner, K., Clark, J. W., Shaw, A. T. (2012). Activity and safety of crizotinib in patients with *ALK*‐positive non‐small‐cell lung cancer: Updated results from a phase 1 study. *Lancet Oncology*, *13*(10), 1011–1019.

68 Jännne, P. A., Yang, J. C‐H., Kim, D‐W., Planchard, D., Ohe, Y., Ramalingam, S. S., Ahn, M‐J., Kim, S‐W., Su, W‐C., Horn, L., Haggstrom, D., Felip, E., Kim, J‐H., Frewer, P., Cantarini, M., Brown, K.H., Dickinson, P. A., Ghiorghiu, S., & Ranson, M. (2015). AZD9291 in EGFR inhibitor‐resistant non‐small‐cell lung cancer. *The New England Journal of Medicine*, *372*(18), 1689–1699.

69 Manji, A., Brana, I., Amir, E., Tomlinson, G., Tannock, I. F., Bedard, P. L., Oza, A., Siu, L. L., Razak, A. R. A. (2013). Evolution of clinical trial design in early drug development: Systematic review of expansion cohort use in single‐agent phase I cancer trials. *Journal of Clinical Oncology*, *31*(33), 4260–4267.

70 Prowell, T. M., Theoret, M. R., & Pazdur, R. (2016). Seamless oncology drug‐development. *The New England Journal of Medicine*, *374*(21), 2001–2003; Hobbs, B. P., Barata, P. C., Kanjanapan, Y., Paller, C. J., Perlmutter, J., Pond, G. R., Prowell, T. M., Rubin, E. H., Seymour, L. K., Wages, N. A., Yap, T. A., Feltquate, D., Garrett‐Mayer, E., Grossman, W., Hong, D. S. Ivy, S. P., Siu, L. L., Reeves, S. A., & Rosner, G. L. (2019). Seamless designs: Current practice and considerations for early‐phase drug development in oncology. *Journal of the National Cancer Institute*, *111*(2), 118–128.

71 Kurzrock, op. cit. note 51, pp. e133–e144.

72 Mackley, op. cit. note 60, pp. 17–26.

73 Park, J. J. H., Siden, E., Zoratti, M. J., Dron, L., Harari, O., Singer, J., Lester, R. T., Thorlund, K., & Mills E. J. (2019). Systematic review of basket trials, umbrella trials, and platform trials: A landscape analysis of master protocols. *Trials*, *20*(1), 572, 1–10.

74 Ibid.

75 MRC Clinical Trials Unit. (2017). *STAMPEDE (Systemic Therapy in Advancing or Metastatic Prostate cancer: Evaluation of Drug Efficacy)*. http://www.stampedetrial.org

76 Sydes, M. R., Parmar, M. K. B., Mason, M. D., Clarke, N. W., Amos, C., Anderson, J., de Bonon, J., Dearnaley, D. P., Swyer, J., Green, C., Jovic, G., Ritchie, A. W. S., Russell, M. J., Sanders, K., Thalmann, G., & James, N. D. (2012). Flexible trial design in practice—Stopping arms for lack‐of‐benefit and adding research arms mid‐trial in STAMPEDE: a multi‐arm multi‐stage randomized controlled trial. *Trials*, *13*(1), 168, 1–14.

77 Sydes, M. R., Parmar, M. K., James, N. D., Clarke, N. W., Dearnaley, D. P., Mason, M. D., Morgan, R. C>, Sanders, K., & Royston, P. (2009). Issues in applying multi‐arm multi‐stage methodology to a clinical trial in prostate cancer: The MRC STAMPEDE trial. *Trials*, *10*(39), 1–16; Morrell, L., Hordern, J., Brown, L., Sydes, M. R., Amos, C. L., Kaplan, R. S., Parar, M. L. B., & Maughan, T. S. (2019). Mind the gap? The platform trial as a working environment. *Trials*, *20*(1), 297, 1–6.

78 Sydes, op. cit. note 76, 168, pp. 1–14; Millen, G. C., & Yap, C. (2020). Adaptive trial designs: What are multiarm, multistage trials? *Archives of Disease in Childhood: Education & Practice*, *105*(6), 376–378.

79 Lin, R., & Lee, J. J. (2020). Novel bayesian adaptive designs and their applications in cancer clinical trials. Computational and Methodological Statistics and Biostatistics, 395–426. https://doi.org/10.1007/978-3-030-42196-0_17

80 Kim, E. S., Herbst, R. S., Wistuba, I., Lee, J. J., Blumenschein, G. R. Jr., Tsao, A., Stewart, D. J., Hicks, M. E., Erasmus Jr, J., Gupta, S., Alden, C. M., Liu, S., Tang, X., Khuri, F. R., Tran, H. T., Johnson, B. E., Heymach, J. V., Mao, L., Fossella, F., Kies, M. S., Papadimitrakopoulou, V., Davis, S. E., Lippman, S. M., & Hong, W. K. (2011). The BATTLE trial: Personalizing therapy for lung cancer. *Cancer Discovery*, *1*(1), 44–53.

81 Wang, H., & Yee, D. (2019). I‐SPY2: A neoadjuvant adaptive clinical trial designed to improve outcomes in high‐risk breast cancer. *Current Breast Cancer Reports*, *11*(4), 303–310.

82 Ibid.

83 Ibid.

84 Ibid.

85 Strzebonska, K., Blukacz, M., Wasylewski, M. T., Polak, M., Gyawali, B., & Waligora, M. (2022). Risk and benefit for umbrella trials in oncology: a systematic review and meta‐analysis. *BMC Medicine*, *20*(1), 219, 1–16; Hazim, A., & Prasad, V. (2018). A pooled analysis of published, basket trials in cancer medicine. *European Journal of Cancer*, *101*, 244–250.

86 Schwaederle, M., Zhao, M., Lee, J. J., Eggermont, A. M., Schilsky, R. L., Mendelsohn, J., Lazar V., & Kurzrock R. (2015). Impact of precision medicine in diverse cancers: A meta‐analysis of phase II clinical trials. *Journal of Clinical Oncology*, *33*(32), 3817–3825.

87 Strzebonska, op. cit. note 84, 219, pp. 1–16.

88 Silvestris, N., Ciliberto, G., De Paoli, P., Apolone, G., Lavitrano, M.L., Pierotti, M.A., Stanta, G., & On the Behalf of the “dynamic medicine OECI group” (2017). Liquid dynamic medicine and N‐of‐1 clinical trials: A change of perspective in oncology research. *Journal of Experimental & Clinical Cancer Research*, *36*(1), 128, 1–5.

89 Gouda, M. A. (2023). N‐of‐1 trials in cancer drug development. *Cancer Discovery*, *13*(6), 1301–1309.

90 Kim, op. cit. note 79, pp. 44–53; Wang, op. cit. note 80, pp. 303–310.

91 Weir, I. R., Marshall, G. D., Schneider, J. I., Sherer, J. A., Lord, E. M., Gyawali, B., Paasche‐Orlow, m. K., Benjamin, E. J., & Trinquart, L. (2019). Interpretation of time‐to‐event outcomes in randomized trials: an online randomized experiment. *Annals of Oncology*, *30*(1), 96–102.

92 Jansen (2016), op. cit. note 23, pp. 1238–1246; Jansen (2017), op. cit. note 23, pp. 280–288.

93 Abernethy, op. cit. note 7, pp. 3794–3800; Pentz, op. cit. note 8, pp. 4571–4578; Campbell, op. cit. note 28, pp. 18–25.

94 Tan, A. C., Ashley, D. M., & Khasraw, M. (2020). Adapting to a pandemic—Conducting oncology trials during the SARS‐CoV‐2 pandemic. *Clinical Cancer Research*, *26*(13), 3100–3103.

95 Lee, J. J., Burbury, K., Underhill, C., Harris, S., Shackleton, K., McBurnie, J., McPhee, N., Osmond, F., Wilkins, K., Baden, P., & Krishnasamy, M. (2022). Exploring Australian regional cancer patients’ experiences of clinical trial participation via telehealth. *Journal of Telemedicine and Telecare*, *28*(7), 508–516; Sabesan, S., Zalcberg, J., Underhill, C., Zielinski, R., Burbury, K., Ansari, Z., Rainey, N., Collins, I., Osbourne, R., Sanmugarajah, J., Joshua, A., & Honeyball, F. (2019). Implementation of the Australasian Teletrial Model: Lessons from practice. *Asia‐Pacific Journal of Clinical Oncology*, *15*(S8), 3–14.

96 National Comprehensive Cancer Network®, op. cit. note 37; Weber, op. cit. note 38, pp. 278–284; Joffe, S., & Weeks, J. C. (2002). Views of American oncologists about the purposes of clinical trials. *Journal of the National Cancer Institute*, *94*(24), 1847–1853.

97 Shumway, op. cit. note 7, pp. 867–875; Weber, op. cit. note 38, pp. 278–284.

98 Hordern, J., Maughan, T., Feiler, T., Morrell, L., Horne, R., & Sullivan, R. (2017). The ‘molecularly unstratified’ patient: A focus for moral, psycho‐social and societal research. *Biomed Hub*, *2*(Suppl 1), 146–153.

99 Mackley, op. cit. note 60, pp. 17–26.

100 Emanuel, E. J., Wendler, D., & Grady, C. (2000). What makes clinical research ethical? *Journal of the American Medical Association*, *283*(20), 2701–2711.

101 Gyawali, B., & Niraula, S. (2020). Lessons from adaptive randomization: Spying the I‐SPY2 trial in breast cancer. *Journal National Comprehensive Cancer Network*, *18*(11), 1441–1444.

102 Seruga, B., Ocana, A., Amir, E., Tannock, I. F. (2015). Failures in phase III: Causes and consequences. *Clinical Cancer Research*, *21*(20), 4552–4560.

103 Saxman, S. B. (2015). Ethical considerations for outcome‐adaptive trial designs: A clinical researcher's perspective. *Bioethics*, *29*(2), 59–65.

104 London, A. J. (2018). Learning health systems, clinical equipoise, and the ethics of response adaptive randomisation. *Journal of Medical Ethics*, *44*(6), 409–415.

105 Joffe, op. cit. note 6, pp. 30–42.

106 Gyawali, B., de Vries, E. G. E., Dafni, U., Amaral, T., Barriuso, J., Bogaerts, J., Calles, A., Curigliano, G., Gomez‐Roca, C., Kiesewetter, B., Oosting, S., Passaro, A., Pentheroudakis, G., Piccart, M., Roitberg, F., Tabernero, J., Tarazona, N., Trapani, D., Wester, R., Zarkavelis, G., Zielinski, C., Zygoura, P., & Cherny, N. I. (2021). Biases in study design, implementation, and data analysis that distort the appraisal of clinical benefit and ESMO‐Magnitude of Clinical Benefit Scale (ESMO‐MCBS) scoring. *ESMO Open*. *6*(3), 100117, 1–11.

107 Emanuel, op. cit. note 99, pp. 2701–2711.

108 Patel, M. I., Lopez, A. M., Blackstock, W., Reeder‐Hayes, K., Moushey, E. A., Phllips, J., & Tap, W. (2020). Cancer disparities and health equity: A policy statement From the American Society of Clinical Oncology. *Journal of Clinical Oncology*, *38*(29), 3439–3448; Pierce, L. (2021). ASCO's commitment to addressing equity, diversity, and inclusion of black cancer patients and survivors. *Journal of Clinical Oncology—Oncology Practice*, *17*(5), 255–257.

109 Penel, N., Lebellec, L., & Vanseymortier, M. (2018). Reappraisal of eligibility criteria in cancer clinical trials. *Current Opinions Oncology*, *30*(5), 352–357.

110 Kimmelman, op. cit. note 24, pp. 36–42.

111 Segelov, E., Tattersall, M. H. N., & Coates, A. S. (1992). Special article: Redressing the balance—The ethics of not entering an eligible patient on a randomised clinical trial: Point of view. *Annals of Oncology*, *3*(2), 103–105.

112 Sisk, B. A., DuBois, J., Hobbs, B. P., Kodish, E. (2019). Reprioritizing risk and benefit: The future of study design in early‐phase cancer research. *Ethics Human Research*, *41*(6), 2–11.

113 Seruga, op. cit. note 101, pp. 4552–4560.

114 Gyawali, B., Rome, B. N., Kesselheim, A. S. (2021). Regulatory and clinical consequences of negative confirmatory trials of accelerated approval cancer drugs: Retrospective observational study. *British Medical Journal*, *374*, n1959.

115 Ibid.

116 Emanuel, op. cit. note 99, pp. 2701–2711.

117 Hey, S., & Kimmelman, J. (2015). Are outcome‐adaptive allocation trials ethical? *Clinical Trials*. 12(2), 102–106.

118 Ibid.

119 Goncharov, L., Suominen, H., & Cook, M. (2022). Dynamic consent and personalised medicine. *Medical Journal of Australia*, *216*(11), 547–549.

120 Haas, M. A, Teare, H., Prictor, M., Ceregra, G., Vidgen, M.E., Bunker, D., Kaye, J., & Boughtwood, T. (2021). ‘CTRL’: An online, dynamic consent and participant engagement platform working towards solving the complexities of consent in genomic research. *European Journal of Human Genetics*, *29*(4), 687–698.

121 Ibid.

122 Christopher, op. cit. note 31, p. e0184224.

123 World Medical Association. (2013). Declaration of Helsinki: Ethical principles for medical research involving human subjects. *Journal of the American Medical Association*, *310*(20), 2191–2194.

124 Darsaut, T. E., & Raymond, J. (2021). Ethical care requires pragmatic care research to guide medical practice under uncertainty. *Trials*, *22*(1), 143, 1–11.

125 Kimmelman, J. (2008). Twenty‐five years of the therapeutic misconception‐Jonathan Kimmelman replies. *The Hastings Center Report*, *38*(2), 6–7.

126 Sisk, op. cit. note 111, pp. 2–11.

