# Supplementary material for: Therapeutic misunderstandings in modern research
Source: Bioethics. 2023 Dec 19;38(2):138–52. doi: 10.1111/bioe.13241 (PMC10952669; doi:10.1111/bioe.13241)
Supplement: Supplementary file 1 — Supporting information. [file BIOE-38-138-s001.docx]

**Supplementary Material: Retrospective studies which have reviewed benefits and risks of participation in phase I cancer trials**

| **Study** | **Time Period** | **Study Methodology** | **Trial Results** | **Measure of Benefit** | **Measure of Toxicity** |
| --- | --- | --- | --- | --- | --- |
| Estey E et al, Cancer Treatment Reports, 1986^1^ | ~1974-1982 | Review of NCI-sponsored phase I cancer trials (1974-1982, 42 cytotoxic agents), additional 12 agents entering clinical trials before 1974  **Tumour types:** solid and haematological tumours  **Participant age:** adults  **Investigational agent types:** Single agent cytotoxics | 187 trials  54 agents  6, 447 participants  **Trial size:** median 25 participants/trial (1980 onwards), 30 participants/trial (1974-1979), 40 participants/trial (pre-1974) | Objective response (CR+PR): 4.2% | **Toxic death rate:** not specified |
| Roberts T et al, JAMA, 2004^2^ | 1991-2002 | Review of ASCO conference abstracts reporting on phase I cancer trials, and associated manuscripts  **Tumour types:** solid tumours  **Participant age:** not-specified  **Investigational agent types:** Single agents without FDA approval for any indication at time of submission to ASCO conference | 213 trials  149 novel agents  6, 474 participants  **Trial size:** mean 30 participants/trial | Overall objective response rate (CR+PR): 3.8% | **Toxic death rate:** 0.54% |
| Horstmann E et al, NEJM, 2005^3^ | 1991-2002 | Review of phase I trials sponsored by the CTEP and conducted at NIH and other US institutions  **Tumour types:** solid and haematological tumours  **Participant age:** adults  **Investigational agent types:** single agent, multiple agents, novel+FDA approved agent, FDA approved agents | 460 trials  11, 935 participants (10,402 participants assessed for response)  **Trial size:** not specified | Overall response rate (CR+PR) = 10.6%  Disease-specific trials: 19.3%  Not disease-specific trials: 6.3%  FIH trials: 4.8%  Non-FIH trials: 13.1% | **Toxic death rate:** 0.49%  FIH trials toxic death rate: 0.26%  Non-FIH toxic death rate: 0.58% |
| Italiano A et al, Annals of Oncology, 2008^4^ | 2003-2006 | Review of all phase I cancer trials conducted at Institut Gustave Roussy  **Tumour type:** solid tumours  **Participant age:** adults  **Investigational agent types:** single agent, novel agent+EMEA-approved agent | 10 trials  180 participants  **Trial size:** not specified | Overall response rate (CR+PR) 7.2%  Disease control rate (objective response + stable disease) 48.2% | **Toxic death rate:** 0.5% |
| Zhang SX et al, JNCI, 2020^5^ | 2005-2010  NB. 1^st^ January 2019 last date for assessing approval status | Review of trials on clinicaltrials.gov and calculation of the ‘therapeutic proportion’*  **Tumour type:** solid and haematological tumours  **Participant age:** not specified  **Investigational agent types:** single agent, multiple agents, novel agents and agents with prior approval being evaluated for different clinical indication | 1,000 trials  922 unique agents or agent combinations (targeted therapy 576/62.5%, cytotoxic 158/17.1%, immunotherapy 129/14.0%, other 59/6.4%)  55 different cancer indications  35,582 participants  **Trial size:** median 26 participants/trial (excluded trials of >200 participants) | 48/922 (5.2%) trials led to FDA approval  31/396 (7.8%) novel drugs received approval  **Therapeutic proportion:** 1.2% (95% CI 1.1-1.3%)   - Not indication specific: 4.9% - NCCN guideline concordant: 3.6%, 12.2% treatment for any disease regardless of dosage   386 patients (1.2%, 95% CI 1.1-1.3%) received ‘therapeutic regimen’ or 1 in every 83 participants enrolled | |
| Mackley MP et al, JCO Precision Oncology, 2021^6^ | 1^st^ January 2015-1^st^ July 2018 | Review of English manuscripts or clinicaltrials.gov entries reporting results of phase I cancer trials  **Tumour type:** solid and haematologic tumours  **Participant age:** adults and paediatric  **Investigational agent types:** single-agent targeted therapies** | 158 trials  6,707 participants (5,582 evaluable for response, 6,442 for toxicity)  **Trial size:** 53/158 (33.5%) included an expansion cohort. 119/158 (75.3%) included </= 50 participants/trial, 32/158 (20.3%) 51-100 participants/trial, and 7/158 (4.4%) >100 participants/trial | Objective response (CR+PR) 6.4% (95% CI 4.6-8.6) | **Toxic death rate:** 0.0% (95% CI 0.0-0.1) |
| Chihara D et al, The Lancet, 2022^7^ | 1^st^ January 2000-31^st^ May 2019 | Review of patient-level data from CTEP-sponsored, investigator-initiated phase 1 trials of patients with solid tumours  **Tumour type:** mostly solid tumours only (90%)  **Participant age:** adults  **Investigational agent types:** single-agent, novel agent+FDA-approved agent, FDA-approved agent for new clinical indication | 465 trials  261 agents  13,847 participants  **Trial size:** median 24 participants/trial | Overall response rate 12.2% (95% CI 11.5-12.8) | **Toxic death rate:** 0.7% (95% CI 0.5-0.8) |

AE = Adverse Event (eg., G4 AE = Grade IV adverse event)

ASCO = American Society of Clinical Oncology

CR = Complete Response on imaging per RECIST guidelines

CTEP = Cancer Therapy Evaluation Program (US)

EMEA = European Medicines Evaluation Agency

FDA = Federal Drug Administration (US)

JAMA = Journal of the American Medical Association

JCO = Journal of Clinical Oncology

JNCI = Journal of the National Cancer Institute

NEJM = New England Journal of Medicine

NIH = National Institutes of Health (NIH)

PR = Partial Response on imaging per RECIST guidelines.

*‘Therapeutic regimen’ was defined by the authors as ‘involving a drug, indication, and approximate dose that received an FDA label, allowing for dose reductions’. The ‘therapeutic proportion’ was defined as the ‘ratio of the total number of patients achieving the outcome of having received a therapeutic regimen to the total number of patients who participated in phase I trials’.^5^

**Agents included ‘targeted immunomodulators, therapies that target specific receptor or signal transduction molecules, and antiangiogenic agents’^6^

1. Estey E, Hoth D, Simon R, et al: Therapeutic response in phase I trials of antineoplastic agents. Cancer Treat Rep 70:1105-15, 1986

2. Roberts TG, Goulart BH, Squitieri L, et al: Trends in the Risks and Benefits to Patients With Cancer Participating in Phase 1 Clinical Trials. JAMA 292:2130-2140, 2004

3. Horstmann E, McCabe MS, Grochow L, et al: Risks and Benefits of Phase 1 Oncology Trials, 1991 through 2002. New England Journal of Medicine 352:895-904, 2005

4. Italiano A, Massard C, Bahleda R, et al: Treatment outcome and survival in participants of phase I oncology trials carried out from 2003 to 2006 at Institut Gustave Roussy. Ann Oncol 19:787-92, 2008

5. Zhang SX, Fergusson D, Kimmelman J: Proportion of Patients in Phase I Oncology Trials Receiving Treatments That Are Ultimately Approved. Journal of the National Cancer Institute 112:886-892, 2020

6. Mackley MP, Fernandez NR, Fletcher B, et al: Revisiting Risk and Benefit in Early Oncology Trials in the Era of Precision Medicine: A Systematic Review and Meta-Analysis of Phase I Trials of Targeted Single-Agent Anticancer Therapies. JCO Precision Oncology:17-26, 2021

7. Chihara D, Lin R, Flowers CR, et al: Early drug development in solid tumours: analysis of National Cancer Institute-sponsored phase 1 trials. The Lancet 400:512-521, 2022
